# ‘Pd_20_Sn_13_’ revisited: crystal structure of Pd_6.69_Sn_4.31_

**DOI:** 10.1107/S2056989015011366

**Published:** 2015-06-17

**Authors:** Wilhelm Klein, Hanpeng Jin, Viktor Hlukhyy, Thomas F. Fässler

**Affiliations:** a Technische Universität München, Department of Chemistry, Lichtenbergstrasse 4, 85747 Garching, Germany

**Keywords:** crystal structure, redetermination, system Pd–Sn, defect variant of the AlB_2_ structure type

## Abstract

The crystal structure of the title compound was previously reported with composition ‘Pd_20_Sn_13_’ [Sarah *et al.* (1981 ▸). *Z. Metallkd*, **72**, 517–520]. For the original structure model, as determined from powder X-ray data, atomic coordinates from the isostructural compound Ni_13_Ga_3_Ge_6_ were transferred. The present structure determination, resulting in a composition Pd_6.69_Sn_4.31_, is based on single crystal X-ray data and includes anisotropic displacement parameters for all atoms as well as standard uncertainties for the atomic coordinates, leading to higher precision and accuracy for the structure model. Single crystals of the title compound were obtained *via* a solid-state reaction route, starting from the elements. The crystal structure can be derived from the AlB_2_ type of structure after removing one eighth of the atoms at the boron positions and shifting adjacent atoms in the same layer in the direction of the voids. One atomic site is partially occupied by both elements with a Pd:Sn ratio of 0.38 (3):0.62 (3). One Sn and three Pd atoms are located on special positions with site symmetry 2. (Wyckoff letter 3*a* and 3*b*).

## Abstract 2

The crystal structure of the title compound, previously reported as ‘Pd_20_Sn_13_’ on basis of powder X-ray data, was redetermined on basis of single-crystal X-ray data, resulting in a model with higher precision and accuracy.

## Chemical context   

In the context of investigations of the binary system Pd—Sn, Nowotny *et al.* (1946 ▸) observed a phase with approximate composition Pd_3_Sn_2_, which was later addressed as ‘Pd_20_Sn_13_’ (Sarah *et al.*, 1981 ▸). According to powder XRD measurements, this compound was found to be isotypical to Ni_13_Ga_3_Ge_6_ (Nover & Schubert, 1981 ▸). Up to now, no further detailed structure examination has been published. In the course of our experiments, aiming at ternary Zintl phases containing tetrel elements (Hlukhyy *et al.*, 2012 ▸), single crystals of the title compound have been obtained in significant amounts and were subjected to a closer structural investigation.

## Structural commentary   

**Figure 1:**
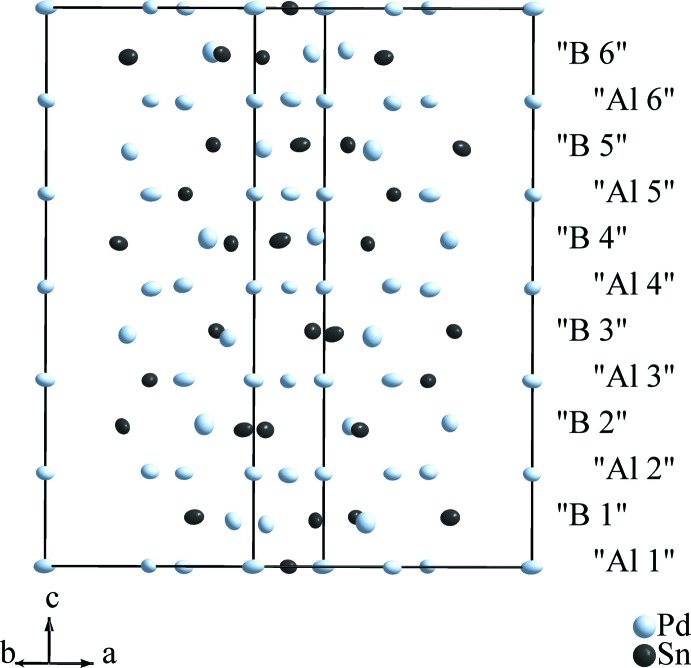
The crystal structure of Pd_6.69_Sn_4.31_, emphasizing the relationship to the AlB_2_ structure type. The ‘Al *n*’ layers represent planes which are occupied by Al atoms in AlB_2_, the ‘B *n*’ layers those with B atoms, respectively. Anisotropic displacement ellipsoids are drawn at the 90% probability level.

The crystal structure of the title compound can be described as a defect variant of the AlB_2_ structure type, where 1/8 of the boron atoms are missing. The symmetry reduction from *P*6/*mmm* to *P*3_2_21 with respect to AlB_2_ results in 13 different crystallographic positions for the Pd and Sn atoms instead of only two, and a more complicated stacking of atomic planes including six differently packed layers for each of the former two, as shown in Fig. 1 ▸. The remaining atomic sites of the B atoms in AlB_2_ are now substituted by seven independent atoms (Pd6, Pd7, Pd8, Sn2, Sn3, Sn4, and Sn5), the ‘Al’ layers are substituted alternatingly by Sn1, Pd3, Pd5, (layers ‘Al1’, ‘Al3’, ‘Al5’ in Fig. 1 ▸), and by Pd1, Pd2, and Pd4 (‘Al2’, ‘Al4’, ‘Al6’), respectively.

**Figure 2:**
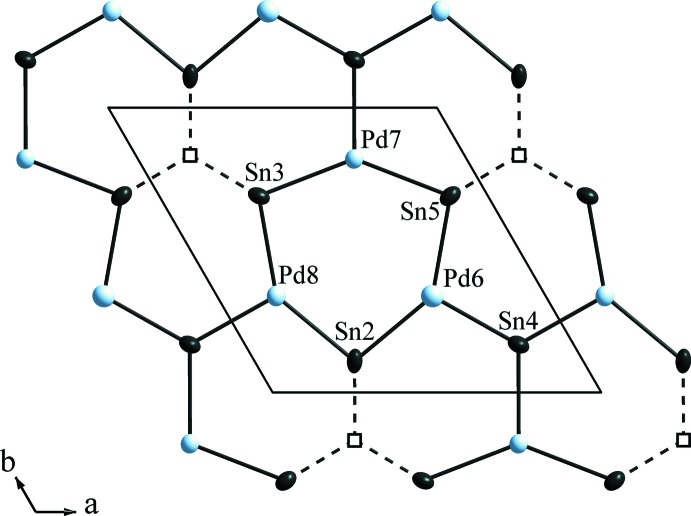
The ‘B1’ layer (see Fig. 1 ▸) in Pd_6.69_Sn_4.31_. To illustrate the relationship to the AlB_2_ structure type, the voids are drawn as empty squares and are connected to the neighbouring Sn atoms by dashed lines. Anisotropic displacement ellipsoids are drawn at the 90% probability level.

The layered character of the Pd_6.69_Sn_4.31_ structure is much less pronounced than in the parent AlB_2_ type of structure, as indicated by the mixed substitution of both the Al and B sites of the AlB_2_ type by Pd as well as by Sn atoms, respectively. Accordingly, there are similar, in average slightly shorter inter­atomic distances within the planes (2.6407 (19) − 2.755 (2) Å) than between them [2.7259 (18)–3.309 (2) Å]. Nevertheless, the layers are clearly distinguishable and only marginally puckered. The distorted honeycomb lattice is obvious if the voids in the ‘B’ layer are considered (Fig. 2 ▸). The distortion results from a shift of the neighbouring Sn atoms within the boron layer (Sn2, Sn3 and Sn5) in the direction of the voids.

**Figure 3:**
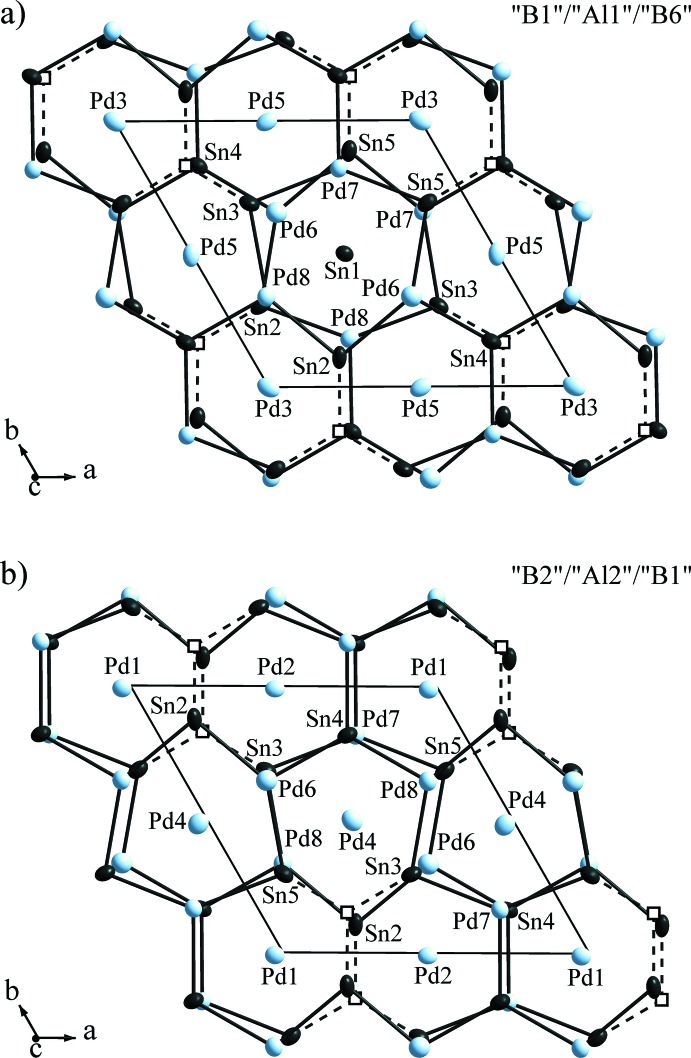
Sections of the crystal structure of Pd_6.69_Sn_4.31_, with *a*) layers ‘B1’–‘Al1’–‘B6’ and *b*) layers ‘B2’–‘Al2’–‘B1’. The voids are drawn as empty squares and are connected to the neighbouring Sn atoms by dashed lines. Shown are the surroundings of the ‘B’ layer atoms with zero (Sn1), one (Pd4) and two voids (Pd1, Pd2, Pd3, Pd5). Anisotropic displacement ellipsoids are drawn at the 90% probability level.

For Sn1 a partial occupation by Pd (Pd9) was found. A full occupation of the (Sn1/Pd9) site (Fig. 3 ▸ *a*) by the element Sn would result in the composition Pd_13_Sn_9_ as suggested by the isostructural compound Ni_13_Ga_3_Ge_6_. However, the occupancy of this position (in contrast to all other Pd and Sn sites) deviates significantly from 100% if only Sn (refined to 96%) or Pd (refined to 107%) is considered. It has to be noticed that this site is the only one in both kinds of ‘Al’ layers that is not close to a void in the ‘B’ layers (Fig. 3 ▸). Consequently, the coordination number (CN) of the (Sn1/Pd9) site is 14, which is higher than that of all other Sn (CN = 10) and Pd atoms (CN = 11–13) in Pd_6.69_Sn_4.31_.

In the previous structure report of ‘Pd_20_Sn_13_’ by Sarah *et al.* (1981 ▸), the atomic parameters were adopted from the isostructural compound Ni_13_Ga_3_Ge_6_, and the occupation of one atomic site was fixed for Sn:Pd as 2/3:1/3. The composition ‘Pd_20_Sn_13_’ was obviously chosen in order to get the indices as integers, however, in consequence *Z* = 2. Our structure refinement suggests a more precise composition Pd_20.06 (5)_Sn_12.94 (5)_. With a crystallographically more appropriate number of formula units, *viz. Z* = 6 (indicating the asymmetric unit), the composition then refined to Pd_6.69 (2)_Sn_4.31 (2)_.

## Synthesis and crystallization   

Single crystals of the title compound were obtained from experiments aiming at a ternary alloy in the chemical system K—Pd—Sn, with similar conditions as reported by Hlukhyy *et al.* (2012 ▸). 23.4 mg K (99.9%, Riedel de Haën), 71 mg Sn (99.999%, ChemPur), and 20.6 mg of PdSn, prefabricated by arc melting of the elements, were filled into a niobium crucible, which was sealed, placed in a silica glass tube, annealed under vacuum for 20 h at 1273 K and subsequently for 72 h at 873 K, and finally quenched with liquid nitro­gen. As a by-product, K_4_Sn_4_ (Hewaidy *et al.*, 1964 ▸) was found.

## Refinement   

Crystal data, data collection and structure refinement details are summarized in Table 1 ▸. In contrast to the previously reported structure model, which was described in *P*3_1_21 (based on powder X-ray data; Sarah *et al.*, 1981 ▸), the crystal under investigation adopts the inverted structure, as indicated by the refined Flack parameter (Flack, 1983 ▸; Parsons *et al.*, 2013 ▸). Therefore space group *P*3_2_21 was chosen for the current refinement. It should be noted that the value and the corresponding standard uncertainty for the Flack parameter are rather high. However, the cause for this behaviour remains unclear. For the Sn1 site a partial occupation by Pd (Pd9) was found, with a refined occupation of 62 (3)% Sn and 38 (3)% Pd. All atoms were refined with anisotropic displacement parameters. The remaining maximum and minimum electron densities are located 2.08 Å from Sn2 and 0.46 Å from Pd8, respectively.

## Supplementary Material

Crystal structure: contains datablock(s) global, I. DOI: 10.1107/S2056989015011366/wm5142sup1.cif

Structure factors: contains datablock(s) I. DOI: 10.1107/S2056989015011366/wm5142Isup2.hkl

CCDC reference: 1406124

Additional supporting information:  crystallographic information; 3D view; checkCIF report

## Figures and Tables

**Table 1:** Experimental details

Crystal data	
Chemical formula	Pd_6.69_Sn_4.31_
*M*_r_	1223.37
Crystal system, space group	Trigonal, *P*3_2_21
Temperature (K)	150
*a*, *c* ()	8.77574(17), 16.9004(4)
*V* (^3^)	1127.18(5)
*Z*	6
Radiation type	Mo *K*
(mm^1^)	29.54
Crystal size (mm)	0.16 0.1 0.08

Data collection	
Diffractometer	Oxford Xcalibur 3
Absorption correction	Multi-scan (*CrysAlis RED*; Oxford Diffraction, 2009 ▸)
*T*_min_, *T*_max_	0.408, 1.000
No. of measured, independent and observed [*I* > 2(*I*)] reflections	20534, 2682, 2001
*R*_int_	0.041
(sin /)_max_ (^1^)	0.762

Refinement	
*R*[*F* ^2^ > 2(*F* ^2^)], *wR*(*F* ^2^), *S*	0.028, 0.076, 1.08
No. of reflections	2682
No. of parameters	104
_max_, _min_ (e ^3^)	2.66, 2.52
Absolute structure	Flack *x* determined using 715 quotients [(*I* ^+^)(*I* )]/[(*I* ^+^)+(*I* )] (Parsons *et al.*, 2013 ▸)
Absolute structure parameter	0.2(2)

Computer programs: *CrysAlis CCD* and *CrysAlis RED* (Oxford Diffraction, 2009 ▸), *SHELXS97* (Sheldrick, 2008 ▸), *SHELXL2014* (Sheldrick, 2015 ▸) and *DIAMOND* (Brandenburg, 2012 ▸).

## supplementary crystallographic information

### Crystal data

**Table:**

Pd_6.69_Sn_4.31_	*D*_x_ = 10.813 Mg m^−^^3^
*M**_r_* = 1223.37	Mo *K*α radiation, λ = 0.71073 Å
Trigonal, *P*3_2_21	Cell parameters from 8247 reflections
*a* = 8.77574 (17) Å	θ = 2.9–32.7°
*c* = 16.9004 (4) Å	µ = 29.54 mm^−^^1^
*V* = 1127.18 (5) Å^3^	*T* = 150 K
*Z* = 6	Fragment, black
*F*(000) = 3139	0.16 × 0.1 × 0.08 mm

### Data collection

**Table:**

Oxford Xcalibur 3 diffractometer	2682 independent reflections
Radiation source: Enhance (Mo) X-ray Source	2001 reflections with *I* > 2σ(*I*)
Graphite monochromator	*R*_int_ = 0.041
Detector resolution: 16.0238 pixels mm^-1^	θ_max_ = 32.8°, θ_min_ = 2.9°
ω and π scans	*h* = −13→8
Absorption correction: multi-scan (*CrysAlis RED*; Oxford Diffraction, 2009)	*k* = −12→12
*T*_min_ = 0.408, *T*_max_ = 1.000	*l* = −25→25
20534 measured reflections	

### Refinement

**Table:**

Refinement on *F*^2^	*w* = 1/[σ^2^(*F*_o_^2^) + (0.0364*P*)^2^ + 1.004*P*] where *P* = (*F*_o_^2^ + 2*F*_c_^2^)/3
Least-squares matrix: full	(Δ/σ)_max_ < 0.001
*R*[*F*^2^ > 2σ(*F*^2^)] = 0.028	Δρ_max_ = 2.66 e Å^−^^3^
*wR*(*F*^2^) = 0.076	Δρ_min_ = −2.52 e Å^−^^3^
*S* = 1.08	Extinction correction: *SHELXL2014* (Sheldrick, 2015), Fc^*^=kFc[1+0.001xFc^2^λ^3^/sin(2θ)]^-1/4^
2682 reflections	Extinction coefficient: 0.00066 (4)
104 parameters	Absolute structure: Flack *x* determined using 715 quotients [(I+)-(I-)]/[(I+)+(I-)] (Parsons *et al.*, 2013)
0 restraints	Absolute structure parameter: −0.2 (2)

### Special details

**Table:**

Geometry. All e.s.d.'s (except the e.s.d. in the dihedral angle between two l.s. planes) are estimated using the full covariance matrix. The cell e.s.d.'s are taken into account individually in the estimation of e.s.d.'s in distances, angles and torsion angles; correlations between e.s.d.'s in cell parameters are only used when they are defined by crystal symmetry. An approximate (isotropic) treatment of cell e.s.d.'s is used for estimating e.s.d.'s involving l.s. planes.

### Fractional atomic coordinates and isotropic or equivalent isotropic displacement parameters (Å^2^)

**Table:**

	*x*	*y*	*z*	*U*_iso_*/*U*_eq_	Occ. (<1)
Pd1	−0.0002 (5)	0.0000	0.1667	0.0083 (2)	
Pd2	0.4990 (2)	0.0000	0.1667	0.00772 (19)	
Pd3	−0.0010 (5)	−0.0010 (5)	0.0000	0.0100 (2)	
Pd4	0.5041 (2)	0.5072 (3)	0.16345 (4)	0.01021 (19)	
Pd5	0.4963 (2)	−0.0035 (5)	−0.00092 (4)	0.00921 (17)	
Pd6	0.6556 (3)	0.3385 (3)	0.07906 (6)	0.0124 (2)	
Pd7	0.6590 (3)	0.82039 (16)	0.07559 (5)	0.00882 (17)	
Pd8	0.18110 (16)	0.3394 (3)	0.08142 (5)	0.00947 (16)	
Pd9	0.4997 (2)	0.4997 (2)	0.0000	0.0071 (3)	0.37 (4)
Sn1	0.4997 (2)	0.4997 (2)	0.0000	0.0071 (3)	0.63 (4)
Sn2	0.3048 (2)	0.11037 (11)	0.08285 (5)	0.00898 (19)	
Sn3	0.3030 (3)	0.6900 (3)	0.08831 (5)	0.0083 (2)	
Sn4	0.83212 (15)	0.16829 (14)	0.08857 (4)	0.00861 (14)	
Sn5	0.88531 (10)	0.6896 (2)	0.08862 (5)	0.0084 (2)	

### Atomic displacement parameters (Å^2^)

**Table:**

	*U*^11^	*U*^22^	*U*^33^	*U*^12^	*U*^13^	*U*^23^
Pd1	0.0096 (8)	0.0087 (14)	0.0063 (5)	0.0044 (7)	0.0006 (5)	0.0012 (11)
Pd2	0.0096 (7)	0.0076 (14)	0.0052 (4)	0.0038 (7)	−0.0002 (5)	−0.0003 (10)
Pd3	0.0104 (7)	0.0104 (7)	0.0062 (5)	0.0028 (13)	0.0005 (6)	−0.0005 (6)
Pd4	0.0100 (8)	0.0122 (6)	0.0072 (3)	0.0046 (8)	0.0002 (6)	0.0011 (4)
Pd5	0.0143 (8)	0.0093 (7)	0.0060 (3)	0.0074 (5)	−0.0004 (5)	−0.0002 (6)
Pd6	0.0116 (8)	0.0116 (8)	0.0137 (4)	0.0056 (5)	0.0001 (6)	0.0005 (6)
Pd7	0.0087 (7)	0.0089 (5)	0.0096 (3)	0.0049 (6)	0.0004 (6)	0.0007 (4)
Pd8	0.0091 (5)	0.0101 (7)	0.0099 (3)	0.0053 (6)	−0.0008 (4)	0.0005 (6)
Pd9	0.0070 (6)	0.0070 (6)	0.0060 (4)	0.0026 (11)	−0.0003 (5)	0.0003 (5)
Sn1	0.0070 (6)	0.0070 (6)	0.0060 (4)	0.0026 (11)	−0.0003 (5)	0.0003 (5)
Sn2	0.0081 (7)	0.0138 (4)	0.0076 (3)	0.0074 (7)	0.0014 (5)	0.0019 (3)
Sn3	0.0080 (7)	0.0069 (7)	0.0070 (4)	0.0015 (3)	0.0001 (5)	−0.0004 (5)
Sn4	0.0079 (4)	0.0072 (4)	0.0081 (3)	0.0018 (4)	0.0002 (3)	−0.0001 (3)
Sn5	0.0123 (4)	0.0089 (7)	0.0065 (3)	0.0070 (7)	−0.0009 (2)	−0.0008 (5)

### Geometric parameters (Å, º)

**Table:**

Pd1—Sn5^i^	2.7259 (18)	Pd5—Pd8^ix^	2.933 (3)
Pd1—Sn5^ii^	2.7259 (18)	Pd5—Sn4	2.967 (2)
Pd1—Sn2^iii^	2.741 (4)	Pd6—Sn4	2.6407 (19)
Pd1—Sn2	2.741 (4)	Pd6—Sn2	2.707 (2)
Pd1—Pd3	2.8168 (1)	Pd6—Sn5	2.715 (2)
Pd1—Pd3^iv^	2.8168 (1)	Pd6—Pd9	2.7553 (13)
Pd1—Sn4^v^	2.875 (3)	Pd6—Sn3^viii^	2.8242 (13)
Pd1—Sn4^vi^	2.875 (3)	Pd6—Sn3^ix^	2.8782 (13)
Pd1—Pd8	2.9563 (18)	Pd6—Pd5^xv^	2.910 (4)
Pd1—Pd8^iii^	2.9563 (18)	Pd6—Pd4^viii^	3.017 (4)
Pd1—Pd7^ii^	3.015 (4)	Pd7—Sn4^xvi^	2.6532 (16)
Pd1—Pd7^i^	3.015 (4)	Pd7—Sn3	2.746 (3)
Pd2—Sn3^vii^	2.7264 (19)	Pd7—Pd9	2.7515 (19)
Pd2—Sn3^viii^	2.7264 (19)	Pd7—Sn5	2.755 (2)
Pd2—Sn2^iii^	2.738 (2)	Pd7—Sn5^ix^	2.8188 (11)
Pd2—Sn2	2.738 (2)	Pd7—Sn4^ii^	2.8603 (9)
Pd2—Pd5	2.8325 (7)	Pd7—Pd5^xvi^	2.882 (3)
Pd2—Pd5^iii^	2.8325 (7)	Pd7—Pd3^xvii^	2.884 (4)
Pd2—Sn4^iii^	2.8554 (19)	Pd7—Pd2^xvi^	3.006 (2)
Pd2—Sn4	2.8554 (19)	Pd7—Pd1^xvii^	3.014 (4)
Pd2—Pd6	2.9705 (19)	Pd8—Sn4^vi^	2.6552 (18)
Pd2—Pd6^iii^	2.9705 (19)	Pd8—Sn3	2.708 (3)
Pd2—Pd7^vii^	3.006 (2)	Pd8—Sn2	2.720 (2)
Pd2—Pd7^viii^	3.006 (2)	Pd8—Sn5^ii^	2.7802 (11)
Pd3—Sn2	2.738 (3)	Pd8—Pd9	2.7853 (18)
Pd3—Sn2^ix^	2.738 (3)	Pd8—Sn2^ix^	2.8279 (11)
Pd3—Sn5^x^	2.811 (3)	Pd8—Pd5^ix^	2.933 (3)
Pd3—Sn5^i^	2.811 (3)	Pd8—Pd4^ii^	2.973 (2)
Pd3—Pd1^xi^	2.8167 (1)	Pd9—Pd7^ix^	2.7515 (19)
Pd3—Pd7^x^	2.884 (4)	Pd9—Pd6^ix^	2.7553 (13)
Pd3—Pd7^i^	2.884 (4)	Pd9—Pd4^ix^	2.7630 (7)
Pd3—Pd8^ix^	2.932 (4)	Pd9—Pd8^ix^	2.7853 (18)
Pd3—Pd8	2.932 (4)	Pd9—Sn2	3.2738 (16)
Pd3—Sn4^vi^	2.9611 (11)	Pd9—Sn2^ix^	3.2738 (16)
Pd3—Sn4^xii^	2.9611 (11)	Sn2—Pd8^ix^	2.8279 (11)
Pd4—Sn3^viii^	2.732 (4)	Sn2—Sn2^iii^	3.2924 (15)
Pd4—Sn5^ii^	2.742 (2)	Sn3—Pd2^xvi^	2.7263 (19)
Pd4—Pd9	2.7629 (7)	Sn3—Pd4^ii^	2.732 (4)
Pd4—Pd7	2.805 (3)	Sn3—Pd5^ix^	2.781 (4)
Pd4—Pd8	2.820 (2)	Sn3—Pd5^xvi^	2.797 (4)
Pd4—Pd6	2.821 (3)	Sn3—Pd6^ii^	2.8242 (13)
Pd4—Sn4^ii^	2.823 (2)	Sn3—Pd6^ix^	2.8783 (13)
Pd4—Pd5^xiii^	2.8558 (9)	Sn4—Pd7^vii^	2.6531 (16)
Pd4—Pd8^viii^	2.973 (2)	Sn4—Pd8^xviii^	2.6552 (18)
Pd4—Pd6^ii^	3.017 (4)	Sn4—Pd4^viii^	2.823 (2)
Pd4—Sn5	3.1621 (19)	Sn4—Pd7^viii^	2.8604 (9)
Pd5—Sn2	2.740 (3)	Sn4—Pd1^xviii^	2.875 (3)
Pd5—Sn5^xii^	2.772 (3)	Sn4—Pd5^xv^	2.900 (2)
Pd5—Sn3^ix^	2.781 (4)	Sn4—Pd3^xviii^	2.9611 (11)
Pd5—Sn3^vii^	2.797 (4)	Sn5—Pd1^xvii^	2.7258 (18)
Pd5—Pd4^xiv^	2.8557 (9)	Sn5—Pd4^viii^	2.742 (2)
Pd5—Pd7^vii^	2.882 (3)	Sn5—Pd5^xv^	2.772 (3)
Pd5—Sn4^xii^	2.900 (2)	Sn5—Pd8^viii^	2.7803 (11)
Pd5—Pd6^xii^	2.910 (4)	Sn5—Pd3^xvii^	2.811 (3)
Pd5—Pd6	2.932 (4)	Sn5—Pd7^ix^	2.8188 (11)
			
Sn5^i^—Pd1—Sn5^ii^	164.96 (17)	Sn2—Pd6—Pd5^xv^	151.78 (6)
Sn5^i^—Pd1—Sn2^iii^	83.14 (7)	Sn5—Pd6—Pd5^xv^	58.92 (6)
Sn5^ii^—Pd1—Sn2^iii^	84.84 (8)	Pd9—Pd6—Pd5^xv^	101.06 (7)
Sn5^i^—Pd1—Sn2	84.84 (8)	Pd4—Pd6—Pd5^xv^	128.45 (10)
Sn5^ii^—Pd1—Sn2	83.14 (7)	Sn3^viii^—Pd6—Pd5^xv^	123.49 (7)
Sn2^iii^—Pd1—Sn2	73.82 (12)	Sn3^ix^—Pd6—Pd5^xv^	57.78 (6)
Sn5^i^—Pd1—Pd3	60.92 (7)	Sn4—Pd6—Pd5	64.09 (5)
Sn5^ii^—Pd1—Pd3	119.10 (9)	Sn2—Pd6—Pd5	57.98 (6)
Sn2^iii^—Pd1—Pd3	121.12 (13)	Sn5—Pd6—Pd5	153.21 (6)
Sn2—Pd1—Pd3	59.00 (9)	Pd9—Pd6—Pd5	101.37 (7)
Sn5^i^—Pd1—Pd3^iv^	119.10 (9)	Pd4—Pd6—Pd5	131.43 (10)
Sn5^ii^—Pd1—Pd3^iv^	60.91 (7)	Sn3^viii^—Pd6—Pd5	113.13 (8)
Sn2^iii^—Pd1—Pd3^iv^	59.00 (9)	Sn3^ix^—Pd6—Pd5	57.19 (6)
Sn2—Pd1—Pd3^iv^	121.11 (13)	Pd5^xv^—Pd6—Pd5	97.37 (4)
Pd3—Pd1—Pd3^iv^	179.9 (2)	Sn4—Pd6—Pd2	60.84 (6)
Sn5^i^—Pd1—Sn4^v^	86.43 (6)	Sn2—Pd6—Pd2	57.45 (7)
Sn5^ii^—Pd1—Sn4^v^	105.28 (8)	Sn5—Pd6—Pd2	144.58 (5)
Sn2^iii^—Pd1—Sn4^v^	103.97 (3)	Pd9—Pd6—Pd2	130.92 (9)
Sn2—Pd1—Sn4^v^	171.20 (4)	Pd4—Pd6—Pd2	99.73 (7)
Pd3—Pd1—Sn4^v^	117.20 (15)	Sn3^viii^—Pd6—Pd2	56.06 (5)
Pd3^iv^—Pd1—Sn4^v^	62.69 (4)	Sn3^ix^—Pd6—Pd2	113.74 (8)
Sn5^i^—Pd1—Sn4^vi^	105.27 (8)	Pd5^xv^—Pd6—Pd2	123.60 (8)
Sn5^ii^—Pd1—Sn4^vi^	86.43 (6)	Pd5—Pd6—Pd2	57.35 (5)
Sn2^iii^—Pd1—Sn4^vi^	171.20 (4)	Sn4—Pd6—Pd4^viii^	59.42 (5)
Sn2—Pd1—Sn4^vi^	103.97 (3)	Sn2—Pd6—Pd4^viii^	145.74 (7)
Pd3—Pd1—Sn4^vi^	62.69 (4)	Sn5—Pd6—Pd4^viii^	56.86 (6)
Pd3^iv^—Pd1—Sn4^vi^	117.20 (15)	Pd9—Pd6—Pd4^viii^	130.73 (10)
Sn4^v^—Pd1—Sn4^vi^	79.49 (11)	Pd4—Pd6—Pd4^viii^	97.46 (5)
Sn5^i^—Pd1—Pd8	120.98 (4)	Sn3^viii^—Pd6—Pd4^viii^	65.93 (5)
Sn5^ii^—Pd1—Pd8	58.42 (3)	Sn3^ix^—Pd6—Pd4^viii^	114.46 (8)
Sn2^iii^—Pd1—Pd8	119.43 (12)	Pd5^xv^—Pd6—Pd4^viii^	57.57 (6)
Sn2—Pd1—Pd8	56.89 (5)	Pd5—Pd6—Pd4^viii^	123.51 (7)
Pd3—Pd1—Pd8	60.99 (8)	Pd2—Pd6—Pd4^viii^	93.11 (6)
Pd3^iv^—Pd1—Pd8	119.01 (8)	Sn4^xvi^—Pd7—Sn3	110.53 (8)
Sn4^v^—Pd1—Pd8	129.67 (13)	Sn4^xvi^—Pd7—Pd9	157.08 (4)
Sn4^vi^—Pd1—Pd8	54.16 (4)	Sn3—Pd7—Pd9	73.74 (5)
Sn5^i^—Pd1—Pd8^iii^	58.42 (3)	Sn4^xvi^—Pd7—Sn5	110.81 (8)
Sn5^ii^—Pd1—Pd8^iii^	120.98 (4)	Sn3—Pd7—Sn5	136.65 (6)
Sn2^iii^—Pd1—Pd8^iii^	56.89 (5)	Pd9—Pd7—Sn5	73.41 (4)
Sn2—Pd1—Pd8^iii^	119.43 (12)	Sn4^xvi^—Pd7—Pd4	143.30 (5)
Pd3—Pd1—Pd8^iii^	119.01 (8)	Sn3—Pd7—Pd4	69.96 (7)
Pd3^iv^—Pd1—Pd8^iii^	60.99 (8)	Pd9—Pd7—Pd4	59.62 (4)
Sn4^v^—Pd1—Pd8^iii^	54.15 (4)	Sn5—Pd7—Pd4	69.31 (7)
Sn4^vi^—Pd1—Pd8^iii^	129.67 (13)	Sn4^xvi^—Pd7—Sn5^ix^	84.66 (4)
Pd8—Pd1—Pd8^iii^	176.07 (17)	Sn3—Pd7—Sn5^ix^	97.76 (7)
Sn5^i^—Pd1—Pd7^ii^	137.27 (13)	Pd9—Pd7—Sn5^ix^	72.42 (3)
Sn5^ii^—Pd1—Pd7^ii^	57.09 (5)	Sn5—Pd7—Sn5^ix^	98.45 (7)
Sn2^iii^—Pd1—Pd7^ii^	117.33 (4)	Pd4—Pd7—Sn5^ix^	132.04 (6)
Sn2—Pd1—Pd7^ii^	135.21 (3)	Sn4^xvi^—Pd7—Sn4^ii^	83.53 (4)
Pd3—Pd1—Pd7^ii^	120.73 (11)	Sn3—Pd7—Sn4^ii^	86.01 (5)
Pd3^iv^—Pd1—Pd7^ii^	59.16 (10)	Pd9—Pd7—Sn4^ii^	119.39 (5)
Sn4^v^—Pd1—Pd7^ii^	53.49 (7)	Sn5—Pd7—Sn4^ii^	86.17 (5)
Sn4^vi^—Pd1—Pd7^ii^	58.06 (7)	Pd4—Pd7—Sn4^ii^	59.77 (4)
Pd8—Pd1—Pd7^ii^	83.04 (6)	Sn5^ix^—Pd7—Sn4^ii^	168.19 (6)
Pd8^iii^—Pd1—Pd7^ii^	99.87 (7)	Sn4^xvi^—Pd7—Pd5^xvi^	64.68 (7)
Sn5^i^—Pd1—Pd7^i^	57.09 (5)	Sn3—Pd7—Pd5^xvi^	59.54 (7)
Sn5^ii^—Pd1—Pd7^i^	137.27 (13)	Pd9—Pd7—Pd5^xvi^	101.87 (7)
Sn2^iii^—Pd1—Pd7^i^	135.21 (3)	Sn5—Pd7—Pd5^xvi^	155.91 (5)
Sn2—Pd1—Pd7^i^	117.33 (4)	Pd4—Pd7—Pd5^xvi^	129.46 (10)
Pd3—Pd1—Pd7^i^	59.16 (10)	Sn5^ix^—Pd7—Pd5^xvi^	58.18 (5)
Pd3^iv^—Pd1—Pd7^i^	120.73 (11)	Sn4^ii^—Pd7—Pd5^xvi^	115.64 (7)
Sn4^v^—Pd1—Pd7^i^	58.06 (7)	Sn4^xvi^—Pd7—Pd3^xvii^	64.50 (7)
Sn4^vi^—Pd1—Pd7^i^	53.49 (7)	Sn3—Pd7—Pd3^xvii^	156.01 (5)
Pd8—Pd1—Pd7^i^	99.87 (7)	Pd9—Pd7—Pd3^xvii^	102.03 (6)
Pd8^iii^—Pd1—Pd7^i^	83.04 (6)	Sn5—Pd7—Pd3^xvii^	59.74 (6)
Pd7^ii^—Pd1—Pd7^i^	86.07 (12)	Pd4—Pd7—Pd3^xvii^	129.03 (9)
Sn3^vii^—Pd2—Sn3^viii^	164.85 (10)	Sn5^ix^—Pd7—Pd3^xvii^	59.05 (6)
Sn3^vii^—Pd2—Sn2^iii^	83.18 (5)	Sn4^ii^—Pd7—Pd3^xvii^	115.50 (7)
Sn3^viii^—Pd2—Sn2^iii^	84.72 (4)	Pd5^xvi^—Pd7—Pd3^xvii^	99.51 (4)
Sn3^vii^—Pd2—Sn2	84.73 (4)	Sn4^xvi^—Pd7—Pd2^xvi^	60.21 (4)
Sn3^viii^—Pd2—Sn2	83.18 (5)	Sn3—Pd7—Pd2^xvi^	56.36 (6)
Sn2^iii^—Pd2—Sn2	73.91 (8)	Pd9—Pd7—Pd2^xvi^	129.97 (8)
Sn3^vii^—Pd2—Pd5	60.38 (8)	Sn5—Pd7—Pd2^xvi^	143.17 (5)
Sn3^viii^—Pd2—Pd5	119.57 (8)	Pd4—Pd7—Pd2^xvi^	96.79 (7)
Sn2^iii^—Pd2—Pd5	120.80 (8)	Sn5^ix^—Pd7—Pd2^xvi^	114.87 (7)
Sn2—Pd2—Pd5	58.89 (6)	Sn4^ii^—Pd7—Pd2^xvi^	58.19 (4)
Sn3^vii^—Pd2—Pd5^iii^	119.57 (8)	Pd5^xvi^—Pd7—Pd2^xvi^	57.46 (4)
Sn3^viii^—Pd2—Pd5^iii^	60.38 (8)	Pd3^xvii^—Pd7—Pd2^xvi^	124.71 (7)
Sn2^iii^—Pd2—Pd5^iii^	58.89 (6)	Sn4^xvi^—Pd7—Pd1^xvii^	60.56 (5)
Sn2—Pd2—Pd5^iii^	120.80 (8)	Sn3—Pd7—Pd1^xvii^	143.39 (6)
Pd5—Pd2—Pd5^iii^	179.68 (12)	Pd9—Pd7—Pd1^xvii^	129.46 (8)
Sn3^vii^—Pd2—Sn4^iii^	86.47 (4)	Sn5—Pd7—Pd1^xvii^	56.17 (7)
Sn3^viii^—Pd2—Sn4^iii^	105.28 (5)	Pd4—Pd7—Pd1^xvii^	96.32 (5)
Sn2^iii^—Pd2—Sn4^iii^	103.61 (3)	Sn5^ix^—Pd7—Pd1^xvii^	115.31 (8)
Sn2—Pd2—Sn4^iii^	171.07 (3)	Sn4^ii^—Pd7—Pd1^xvii^	58.52 (5)
Pd5—Pd2—Sn4^iii^	117.39 (9)	Pd5^xvi^—Pd7—Pd1^xvii^	125.23 (7)
Pd5^iii^—Pd2—Sn4^iii^	62.89 (5)	Pd3^xvii^—Pd7—Pd1^xvii^	57.00 (6)
Sn3^vii^—Pd2—Sn4	105.27 (5)	Pd2^xvi^—Pd7—Pd1^xvii^	93.78 (5)
Sn3^viii^—Pd2—Sn4	86.47 (4)	Sn4^vi^—Pd8—Sn3	109.23 (8)
Sn2^iii^—Pd2—Sn4	171.07 (3)	Sn4^vi^—Pd8—Sn2	110.81 (8)
Sn2—Pd2—Sn4	103.61 (3)	Sn3—Pd8—Sn2	139.66 (6)
Pd5—Pd2—Sn4	62.89 (5)	Sn4^vi^—Pd8—Sn5^ii^	89.76 (5)
Pd5^iii^—Pd2—Sn4	117.39 (9)	Sn3—Pd8—Sn5^ii^	92.94 (6)
Sn4^iii^—Pd2—Sn4	80.13 (7)	Sn2—Pd8—Sn5^ii^	82.52 (5)
Sn3^vii^—Pd2—Pd6	120.09 (4)	Sn4^vi^—Pd8—Pd9	152.99 (4)
Sn3^viii^—Pd2—Pd6	59.25 (4)	Sn3—Pd8—Pd9	73.79 (5)
Sn2^iii^—Pd2—Pd6	119.55 (7)	Sn2—Pd8—Pd9	72.96 (4)
Sn2—Pd2—Pd6	56.43 (4)	Sn5^ii^—Pd8—Pd9	117.14 (6)
Pd5—Pd2—Pd6	60.64 (8)	Sn4^vi^—Pd8—Pd4	147.89 (4)
Pd5^iii^—Pd2—Pd6	119.35 (8)	Sn3—Pd8—Pd4	70.28 (8)
Sn4^iii^—Pd2—Pd6	130.31 (7)	Sn2—Pd8—Pd4	73.34 (8)
Sn4—Pd2—Pd6	53.86 (4)	Sn5^ii^—Pd8—Pd4	58.64 (5)
Sn3^vii^—Pd2—Pd6^iii^	59.25 (4)	Pd9—Pd8—Pd4	59.07 (4)
Sn3^viii^—Pd2—Pd6^iii^	120.09 (4)	Sn4^vi^—Pd8—Sn2^ix^	81.65 (4)
Sn2^iii^—Pd2—Pd6^iii^	56.43 (4)	Sn3—Pd8—Sn2^ix^	96.03 (8)
Sn2—Pd2—Pd6^iii^	119.56 (7)	Sn2—Pd8—Sn2^ix^	94.39 (7)
Pd5—Pd2—Pd6^iii^	119.34 (8)	Sn5^ii^—Pd8—Sn2^ix^	169.22 (8)
Pd5^iii^—Pd2—Pd6^iii^	60.64 (8)	Pd9—Pd8—Sn2^ix^	71.35 (3)
Sn4^iii^—Pd2—Pd6^iii^	53.86 (4)	Pd4—Pd8—Sn2^ix^	130.40 (5)
Sn4—Pd2—Pd6^iii^	130.31 (7)	Sn4^vi^—Pd8—Pd3	63.78 (6)
Pd6—Pd2—Pd6^iii^	175.71 (10)	Sn3—Pd8—Pd3	151.91 (5)
Sn3^vii^—Pd2—Pd7^vii^	56.98 (4)	Sn2—Pd8—Pd3	57.79 (6)
Sn3^viii^—Pd2—Pd7^vii^	137.49 (8)	Sn5^ii^—Pd8—Pd3	113.52 (8)
Sn2^iii^—Pd2—Pd7^vii^	135.11 (4)	Pd9—Pd8—Pd3	100.42 (6)
Sn2—Pd2—Pd7^vii^	117.19 (3)	Pd4—Pd8—Pd3	131.02 (11)
Pd5—Pd2—Pd7^vii^	59.06 (6)	Sn2^ix^—Pd8—Pd3	56.72 (6)
Pd5^iii^—Pd2—Pd7^vii^	121.22 (7)	Sn4^vi^—Pd8—Pd5^ix^	62.28 (7)
Sn4^iii^—Pd2—Pd7^vii^	58.34 (4)	Sn3—Pd8—Pd5^ix^	58.92 (7)
Sn4—Pd2—Pd7^vii^	53.75 (5)	Sn2—Pd8—Pd5^ix^	150.35 (5)
Pd6—Pd2—Pd7^vii^	99.52 (4)	Sn5^ii^—Pd8—Pd5^ix^	124.40 (6)
Pd6^iii^—Pd2—Pd7^vii^	83.64 (5)	Pd9—Pd8—Pd5^ix^	100.63 (7)
Sn3^vii^—Pd2—Pd7^viii^	137.49 (8)	Pd4—Pd8—Pd5^ix^	129.05 (12)
Sn3^viii^—Pd2—Pd7^viii^	56.98 (4)	Sn2^ix^—Pd8—Pd5^ix^	56.76 (5)
Sn2^iii^—Pd2—Pd7^viii^	117.19 (3)	Pd3—Pd8—Pd5^ix^	96.48 (4)
Sn2—Pd2—Pd7^viii^	135.11 (4)	Sn4^vi^—Pd8—Pd1	61.35 (9)
Pd5—Pd2—Pd7^viii^	121.22 (7)	Sn3—Pd8—Pd1	146.27 (5)
Pd5^iii^—Pd2—Pd7^viii^	59.06 (6)	Sn2—Pd8—Pd1	57.57 (9)
Sn4^iii^—Pd2—Pd7^viii^	53.75 (5)	Sn5^ii^—Pd8—Pd1	56.64 (4)
Sn4—Pd2—Pd7^viii^	58.35 (4)	Pd9—Pd8—Pd1	130.41 (10)
Pd6—Pd2—Pd7^viii^	83.64 (5)	Pd4—Pd8—Pd1	100.40 (5)
Pd6^iii^—Pd2—Pd7^viii^	99.52 (4)	Sn2^ix^—Pd8—Pd1	113.05 (7)
Pd7^vii^—Pd2—Pd7^viii^	86.36 (9)	Pd3—Pd8—Pd1	57.15 (5)
Sn2—Pd3—Sn2^ix^	96.07 (14)	Pd5^ix^—Pd8—Pd1	123.62 (12)
Sn2—Pd3—Sn5^x^	178.55 (7)	Sn4^vi^—Pd8—Pd4^ii^	59.90 (7)
Sn2^ix^—Pd3—Sn5^x^	83.31 (2)	Sn3—Pd8—Pd4^ii^	57.26 (8)
Sn2—Pd3—Sn5^i^	83.31 (2)	Sn2—Pd8—Pd4^ii^	146.97 (6)
Sn2^ix^—Pd3—Sn5^i^	178.55 (7)	Sn5^ii^—Pd8—Pd4^ii^	66.59 (3)
Sn5^x^—Pd3—Sn5^i^	97.34 (13)	Pd9—Pd8—Pd4^ii^	130.97 (10)
Sn2—Pd3—Pd1^xi^	120.62 (10)	Pd4—Pd8—Pd4^ii^	98.54 (4)
Sn2^ix^—Pd3—Pd1^xi^	59.12 (10)	Sn2^ix^—Pd8—Pd4^ii^	113.73 (7)
Sn5^x^—Pd3—Pd1^xi^	57.95 (4)	Pd3—Pd8—Pd4^ii^	123.68 (10)
Sn5^i^—Pd3—Pd1^xi^	122.31 (15)	Pd5^ix^—Pd8—Pd4^ii^	57.83 (5)
Sn2—Pd3—Pd1	59.12 (10)	Pd1—Pd8—Pd4^ii^	94.05 (4)
Sn2^ix^—Pd3—Pd1	120.62 (10)	Pd7—Pd9—Pd7^ix^	80.14 (9)
Sn5^x^—Pd3—Pd1	122.32 (15)	Pd7—Pd9—Pd6^ix^	80.90 (7)
Sn5^i^—Pd3—Pd1	57.95 (4)	Pd7^ix^—Pd9—Pd6^ix^	99.86 (4)
Pd1^xi^—Pd3—Pd1	179.7 (2)	Pd7—Pd9—Pd6	99.87 (4)
Sn2—Pd3—Pd7^x^	121.64 (4)	Pd7^ix^—Pd9—Pd6	80.90 (7)
Sn2^ix^—Pd3—Pd7^x^	122.05 (4)	Pd6^ix^—Pd9—Pd6	179.02 (12)
Sn5^x^—Pd3—Pd7^x^	57.85 (8)	Pd7—Pd9—Pd4	61.16 (6)
Sn5^i^—Pd3—Pd7^x^	59.33 (8)	Pd7^ix^—Pd9—Pd4	116.98 (7)
Pd1^xi^—Pd3—Pd7^x^	63.84 (10)	Pd6^ix^—Pd9—Pd4	118.52 (8)
Pd1—Pd3—Pd7^x^	116.45 (11)	Pd6—Pd9—Pd4	61.50 (8)
Sn2—Pd3—Pd7^i^	122.05 (4)	Pd7—Pd9—Pd4^ix^	116.98 (7)
Sn2^ix^—Pd3—Pd7^i^	121.64 (4)	Pd7^ix^—Pd9—Pd4^ix^	61.16 (6)
Sn5^x^—Pd3—Pd7^i^	59.33 (8)	Pd6^ix^—Pd9—Pd4^ix^	61.50 (8)
Sn5^i^—Pd3—Pd7^i^	57.85 (8)	Pd6—Pd9—Pd4^ix^	118.52 (8)
Pd1^xi^—Pd3—Pd7^i^	116.45 (11)	Pd4—Pd9—Pd4^ix^	177.85 (12)
Pd1—Pd3—Pd7^i^	63.84 (10)	Pd7—Pd9—Pd8^ix^	178.02 (4)
Pd7^x^—Pd3—Pd7^i^	75.79 (12)	Pd7^ix^—Pd9—Pd8^ix^	98.94 (3)
Sn2—Pd3—Pd8^ix^	59.72 (8)	Pd6^ix^—Pd9—Pd8^ix^	97.57 (4)
Sn2^ix^—Pd3—Pd8^ix^	57.22 (8)	Pd6—Pd9—Pd8^ix^	81.68 (7)
Sn5^x^—Pd3—Pd8^ix^	118.91 (4)	Pd4—Pd9—Pd8^ix^	120.78 (8)
Sn5^i^—Pd3—Pd8^ix^	123.33 (4)	Pd4^ix^—Pd9—Pd8^ix^	61.08 (5)
Pd1^xi^—Pd3—Pd8^ix^	61.85 (5)	Pd7—Pd9—Pd8	98.94 (3)
Pd1—Pd3—Pd8^ix^	117.85 (15)	Pd7^ix^—Pd9—Pd8	178.02 (4)
Pd7^x^—Pd3—Pd8^ix^	103.57 (3)	Pd6^ix^—Pd9—Pd8	81.69 (7)
Pd7^i^—Pd3—Pd8^ix^	178.21 (6)	Pd6—Pd9—Pd8	97.57 (4)
Sn2—Pd3—Pd8	57.22 (8)	Pd4—Pd9—Pd8	61.08 (5)
Sn2^ix^—Pd3—Pd8	59.72 (8)	Pd4^ix^—Pd9—Pd8	120.78 (8)
Sn5^x^—Pd3—Pd8	123.32 (4)	Pd8^ix^—Pd9—Pd8	82.03 (9)
Sn5^i^—Pd3—Pd8	118.91 (4)	Pd7—Pd9—Sn2	127.01 (2)
Pd1^xi^—Pd3—Pd8	117.85 (15)	Pd7^ix^—Pd9—Sn2	126.63 (6)
Pd1—Pd3—Pd8	61.85 (5)	Pd6^ix^—Pd9—Sn2	126.54 (8)
Pd7^x^—Pd3—Pd8	178.21 (6)	Pd6—Pd9—Sn2	52.49 (6)
Pd7^i^—Pd3—Pd8	103.57 (3)	Pd4—Pd9—Sn2	65.85 (6)
Pd8^ix^—Pd3—Pd8	77.12 (11)	Pd4^ix^—Pd9—Sn2	116.00 (6)
Sn2—Pd3—Sn4^vi^	101.81 (8)	Pd8^ix^—Pd9—Sn2	54.93 (4)
Sn2^ix^—Pd3—Sn4^vi^	77.90 (6)	Pd8—Pd9—Sn2	52.61 (5)
Sn5^x^—Pd3—Sn4^vi^	79.35 (6)	Pd7—Pd9—Sn2^ix^	126.63 (6)
Sn5^i^—Pd3—Sn4^vi^	100.92 (7)	Pd7^ix^—Pd9—Sn2^ix^	127.01 (2)
Pd1^xi^—Pd3—Sn4^vi^	120.39 (7)	Pd6^ix^—Pd9—Sn2^ix^	52.49 (6)
Pd1—Pd3—Sn4^vi^	59.61 (7)	Pd6—Pd9—Sn2^ix^	126.54 (8)
Pd7^x^—Pd3—Sn4^vi^	126.43 (14)	Pd4—Pd9—Sn2^ix^	116.00 (6)
Pd7^i^—Pd3—Sn4^vi^	53.97 (4)	Pd4^ix^—Pd9—Sn2^ix^	65.85 (6)
Pd8^ix^—Pd3—Sn4^vi^	126.04 (13)	Pd8^ix^—Pd9—Sn2^ix^	52.61 (5)
Pd8—Pd3—Sn4^vi^	53.55 (4)	Pd8—Pd9—Sn2^ix^	54.93 (4)
Sn2—Pd3—Sn4^xii^	77.90 (6)	Sn2—Pd9—Sn2^ix^	76.90 (7)
Sn2^ix^—Pd3—Sn4^xii^	101.81 (8)	Pd6—Sn2—Pd8	100.35 (4)
Sn5^x^—Pd3—Sn4^xii^	100.92 (7)	Pd6—Sn2—Pd3	143.25 (7)
Sn5^i^—Pd3—Sn4^xii^	79.35 (6)	Pd8—Sn2—Pd3	64.99 (7)
Pd1^xi^—Pd3—Sn4^xii^	59.61 (7)	Pd6—Sn2—Pd2	66.12 (6)
Pd1—Pd3—Sn4^xii^	120.39 (7)	Pd8—Sn2—Pd2	144.55 (4)
Pd7^x^—Pd3—Sn4^xii^	53.97 (4)	Pd3—Sn2—Pd2	144.15 (7)
Pd7^i^—Pd3—Sn4^xii^	126.43 (14)	Pd6—Sn2—Pd5	65.14 (8)
Pd8^ix^—Pd3—Sn4^xii^	53.56 (4)	Pd8—Sn2—Pd5	144.00 (6)
Pd8—Pd3—Sn4^xii^	126.04 (13)	Pd3—Sn2—Pd5	106.02 (4)
Sn4^vi^—Pd3—Sn4^xii^	179.59 (18)	Pd2—Sn2—Pd5	62.27 (5)
Sn3^viii^—Pd4—Sn5^ii^	84.03 (6)	Pd6—Sn2—Pd1	145.28 (4)
Sn3^viii^—Pd4—Pd9	119.64 (12)	Pd8—Sn2—Pd1	65.54 (7)
Sn5^ii^—Pd4—Pd9	119.22 (7)	Pd3—Sn2—Pd1	61.87 (6)
Sn3^viii^—Pd4—Pd7	137.40 (9)	Pd2—Sn2—Pd1	106.14 (5)
Sn5^ii^—Pd4—Pd7	136.41 (14)	Pd5—Sn2—Pd1	143.76 (8)
Pd9—Pd4—Pd7	59.22 (5)	Pd6—Sn2—Pd8^ix^	81.77 (6)
Sn3^viii^—Pd4—Pd8	119.74 (10)	Pd8—Sn2—Pd8^ix^	82.40 (5)
Sn5^ii^—Pd4—Pd8	59.96 (5)	Pd3—Sn2—Pd8^ix^	63.56 (7)
Pd9—Pd4—Pd8	59.85 (5)	Pd2—Sn2—Pd8^ix^	124.61 (8)
Pd7—Pd4—Pd8	96.87 (7)	Pd5—Sn2—Pd8^ix^	63.55 (6)
Sn3^viii^—Pd4—Pd6	61.11 (8)	Pd1—Sn2—Pd8^ix^	124.30 (10)
Sn5^ii^—Pd4—Pd6	119.88 (11)	Pd6—Sn2—Pd9	53.86 (4)
Pd9—Pd4—Pd6	59.12 (4)	Pd8—Sn2—Pd9	54.43 (3)
Pd7—Pd4—Pd6	97.00 (5)	Pd3—Sn2—Pd9	93.51 (6)
Pd8—Pd4—Pd6	95.27 (6)	Pd2—Sn2—Pd9	119.84 (6)
Sn3^viii^—Pd4—Sn4^ii^	103.82 (5)	Pd5—Sn2—Pd9	93.67 (7)
Sn5^ii^—Pd4—Sn4^ii^	103.15 (8)	Pd1—Sn2—Pd9	119.88 (7)
Pd9—Pd4—Sn4^ii^	120.30 (9)	Pd8^ix^—Sn2—Pd9	53.72 (3)
Pd7—Pd4—Sn4^ii^	61.08 (6)	Pd6—Sn2—Sn2^iii^	110.29 (4)
Pd8—Pd4—Sn4^ii^	128.95 (14)	Pd8—Sn2—Sn2^iii^	109.49 (3)
Pd6—Pd4—Sn4^ii^	130.44 (9)	Pd3—Sn2—Sn2^iii^	106.41 (6)
Sn3^viii^—Pd4—Pd5^xiii^	59.65 (9)	Pd2—Sn2—Sn2^iii^	53.05 (4)
Sn5^ii^—Pd4—Pd5^xiii^	59.32 (6)	Pd5—Sn2—Sn2^iii^	106.48 (6)
Pd9—Pd4—Pd5^xiii^	178.23 (11)	Pd1—Sn2—Sn2^iii^	53.09 (6)
Pd7—Pd4—Pd5^xiii^	122.48 (10)	Pd8^ix^—Sn2—Sn2^iii^	160.33 (5)
Pd8—Pd4—Pd5^xiii^	118.85 (9)	Pd9—Sn2—Sn2^iii^	145.95 (4)
Pd6—Pd4—Pd5^xiii^	120.40 (14)	Pd8—Sn3—Pd2^xvi^	148.03 (5)
Sn4^ii^—Pd4—Pd5^xiii^	61.40 (6)	Pd8—Sn3—Pd4^ii^	66.25 (7)
Sn3^viii^—Pd4—Pd8^viii^	56.49 (6)	Pd2^xvi^—Sn3—Pd4^ii^	108.48 (6)
Sn5^ii^—Pd4—Pd8^viii^	118.66 (5)	Pd8—Sn3—Pd7	101.00 (4)
Pd9—Pd4—Pd8^viii^	120.82 (8)	Pd2^xvi^—Sn3—Pd7	66.65 (7)
Pd7—Pd4—Pd8^viii^	86.42 (5)	Pd4^ii^—Sn3—Pd7	148.98 (6)
Pd8—Pd4—Pd8^viii^	176.19 (13)	Pd8—Sn3—Pd5^ix^	64.58 (6)
Pd6—Pd4—Pd8^viii^	82.38 (9)	Pd2^xvi^—Sn3—Pd5^ix^	143.39 (7)
Sn4^ii^—Pd4—Pd8^viii^	54.45 (5)	Pd4^ii^—Sn3—Pd5^ix^	62.39 (7)
Pd5^xiii^—Pd4—Pd8^viii^	60.38 (7)	Pd7—Sn3—Pd5^ix^	139.85 (6)
Sn3^viii^—Pd4—Pd6^ii^	117.94 (5)	Pd8—Sn3—Pd5^xvi^	140.76 (6)
Sn5^ii^—Pd4—Pd6^ii^	56.01 (6)	Pd2^xvi^—Sn3—Pd5^xvi^	61.69 (6)
Pd9—Pd4—Pd6^ii^	121.00 (11)	Pd4^ii^—Sn3—Pd5^xvi^	144.37 (7)
Pd7—Pd4—Pd6^ii^	86.30 (10)	Pd7—Sn3—Pd5^xvi^	62.65 (6)
Pd8—Pd4—Pd6^ii^	81.61 (8)	Pd5^ix^—Sn3—Pd5^xvi^	103.75 (3)
Pd6—Pd4—Pd6^ii^	175.73 (7)	Pd8—Sn3—Pd6^ii^	87.24 (6)
Sn4^ii^—Pd4—Pd6^ii^	53.63 (7)	Pd2^xvi^—Sn3—Pd6^ii^	64.69 (5)
Pd5^xiii^—Pd4—Pd6^ii^	59.33 (9)	Pd4^ii^—Sn3—Pd6^ii^	61.01 (6)
Pd8^viii^—Pd4—Pd6^ii^	100.58 (5)	Pd7—Sn3—Pd6^ii^	91.36 (5)
Sn3^viii^—Pd4—Sn5	84.60 (8)	Pd5^ix^—Sn3—Pd6^ii^	123.02 (9)
Sn5^ii^—Pd4—Sn5	168.62 (12)	Pd5^xvi^—Sn3—Pd6^ii^	126.05 (9)
Pd9—Pd4—Sn5	67.09 (5)	Pd8—Sn3—Pd6^ix^	80.82 (6)
Pd7—Pd4—Sn5	54.59 (5)	Pd2^xvi^—Sn3—Pd6^ix^	122.33 (9)
Pd8—Pd4—Sn5	126.91 (3)	Pd4^ii^—Sn3—Pd6^ix^	123.59 (10)
Pd6—Pd4—Sn5	53.61 (7)	Pd7—Sn3—Pd6^ix^	78.84 (6)
Sn4^ii^—Pd4—Sn5	79.51 (5)	Pd5^ix^—Sn3—Pd6^ix^	62.38 (7)
Pd5^xiii^—Pd4—Sn5	114.16 (9)	Pd5^xvi^—Sn3—Pd6^ix^	61.68 (7)
Pd8^viii^—Pd4—Sn5	53.79 (4)	Pd6^ii^—Sn3—Pd6^ix^	162.73 (5)
Pd6^ii^—Pd4—Sn5	130.65 (9)	Pd8—Sn3—Pd4	56.50 (5)
Sn2—Pd5—Sn5^xii^	178.71 (10)	Pd2^xvi^—Sn3—Pd4	94.42 (7)
Sn2—Pd5—Sn3^ix^	96.39 (11)	Pd4^ii^—Sn3—Pd4	95.55 (4)
Sn5^xii^—Pd5—Sn3^ix^	82.57 (4)	Pd7—Sn3—Pd4	55.90 (4)
Sn2—Pd5—Sn3^vii^	83.37 (4)	Pd5^ix^—Sn3—Pd4	120.95 (7)
Sn5^xii^—Pd5—Sn3^vii^	97.68 (11)	Pd5^xvi^—Sn3—Pd4	118.51 (7)
Sn3^ix^—Pd5—Sn3^vii^	179.44 (10)	Pd6^ii^—Sn3—Pd4	59.95 (5)
Sn2—Pd5—Pd2	58.84 (6)	Pd6^ix^—Sn3—Pd4	102.87 (5)
Sn5^xii^—Pd5—Pd2	122.37 (9)	Pd6—Sn4—Pd7^vii^	119.05 (5)
Sn3^ix^—Pd5—Pd2	121.51 (11)	Pd6—Sn4—Pd8^xviii^	120.96 (5)
Sn3^vii^—Pd5—Pd2	57.93 (4)	Pd7^vii^—Sn4—Pd8^xviii^	118.82 (5)
Sn2—Pd5—Pd4^xiv^	120.49 (9)	Pd6—Sn4—Pd4^viii^	66.95 (7)
Sn5^xii^—Pd5—Pd4^xiv^	58.30 (5)	Pd7^vii^—Sn4—Pd4^viii^	155.61 (4)
Sn3^ix^—Pd5—Pd4^xiv^	57.96 (9)	Pd8^xviii^—Sn4—Pd4^viii^	65.64 (8)
Sn3^vii^—Pd5—Pd4^xiv^	122.59 (15)	Pd6—Sn4—Pd2	65.30 (6)
Pd2—Pd5—Pd4^xiv^	179.22 (17)	Pd7^vii^—Sn4—Pd2	66.04 (5)
Sn2—Pd5—Pd7^vii^	121.49 (5)	Pd8^xviii^—Sn4—Pd2	155.06 (4)
Sn5^xii^—Pd5—Pd7^vii^	59.78 (6)	Pd4^viii^—Sn4—Pd2	99.92 (6)
Sn3^ix^—Pd5—Pd7^vii^	121.99 (7)	Pd6—Sn4—Pd7^viii^	92.76 (6)
Sn3^vii^—Pd5—Pd7^vii^	57.81 (7)	Pd7^vii^—Sn4—Pd7^viii^	96.47 (4)
Pd2—Pd5—Pd7^vii^	63.48 (6)	Pd8^xviii^—Sn4—Pd7^viii^	91.64 (5)
Pd4^xiv^—Pd5—Pd7^vii^	117.25 (10)	Pd4^viii^—Sn4—Pd7^viii^	59.15 (4)
Sn2—Pd5—Sn4^xii^	78.94 (6)	Pd2—Sn4—Pd7^viii^	63.47 (5)
Sn5^xii^—Pd5—Sn4^xii^	100.48 (7)	Pd6—Sn4—Pd1^xviii^	156.15 (5)
Sn3^ix^—Pd5—Sn4^xii^	100.64 (6)	Pd7^vii^—Sn4—Pd1^xviii^	65.95 (6)
Sn3^vii^—Pd5—Sn4^xii^	79.82 (8)	Pd8^xviii^—Sn4—Pd1^xviii^	64.49 (7)
Pd2—Pd5—Sn4^xii^	121.13 (7)	Pd4^viii^—Sn4—Pd1^xviii^	99.16 (4)
Pd4^xiv^—Pd5—Sn4^xii^	58.75 (6)	Pd2—Sn4—Pd1^xviii^	100.19 (5)
Pd7^vii^—Pd5—Sn4^xii^	126.67 (13)	Pd7^viii^—Sn4—Pd1^xviii^	63.42 (6)
Sn2—Pd5—Pd6^xii^	123.11 (9)	Pd6—Sn4—Pd5^xv^	63.17 (8)
Sn5^xii^—Pd5—Pd6^xii^	57.03 (7)	Pd7^vii^—Sn4—Pd5^xv^	144.54 (4)
Sn3^ix^—Pd5—Pd6^xii^	119.99 (5)	Pd8^xviii^—Sn4—Pd5^xv^	63.56 (8)
Sn3^vii^—Pd5—Pd6^xii^	60.54 (8)	Pd4^viii^—Sn4—Pd5^xv^	59.85 (4)
Pd2—Pd5—Pd6^xii^	117.52 (11)	Pd2—Sn4—Pd5^xv^	128.46 (9)
Pd4^xiv^—Pd5—Pd6^xii^	63.11 (9)	Pd7^viii^—Sn4—Pd5^xv^	119.00 (5)
Pd7^vii^—Pd5—Pd6^xii^	76.17 (9)	Pd1^xviii^—Sn4—Pd5^xv^	128.04 (10)
Sn4^xii^—Pd5—Pd6^xii^	54.07 (6)	Pd6—Sn4—Pd3^xviii^	146.14 (3)
Sn2—Pd5—Pd6	56.88 (7)	Pd7^vii^—Sn4—Pd3^xviii^	61.52 (9)
Sn5^xii^—Pd5—Pd6	122.99 (9)	Pd8^xviii^—Sn4—Pd3^xviii^	62.67 (9)
Sn3^ix^—Pd5—Pd6	60.43 (8)	Pd4^viii^—Sn4—Pd3^xviii^	128.30 (12)
Sn3^vii^—Pd5—Pd6	119.04 (5)	Pd2—Sn4—Pd3^xviii^	127.55 (9)
Pd2—Pd5—Pd6	62.01 (5)	Pd7^viii^—Sn4—Pd3^xviii^	121.10 (6)
Pd4^xiv^—Pd5—Pd6	117.36 (14)	Pd1^xviii^—Sn4—Pd3^xviii^	57.70 (4)
Pd7^vii^—Pd5—Pd6	103.40 (5)	Pd5^xv^—Sn4—Pd3^xviii^	96.58 (4)
Sn4^xii^—Pd5—Pd6	126.25 (10)	Pd6—Sn4—Pd5	62.73 (8)
Pd6^xii^—Pd5—Pd6	179.50 (7)	Pd7^vii^—Sn4—Pd5	61.39 (8)
Sn2—Pd5—Pd8^ix^	59.68 (6)	Pd8^xviii^—Sn4—Pd5	146.72 (4)
Sn5^xii^—Pd5—Pd8^ix^	119.04 (5)	Pd4^viii^—Sn4—Pd5	129.67 (10)
Sn3^ix^—Pd5—Pd8^ix^	56.50 (7)	Pd2—Sn4—Pd5	58.18 (3)
Sn3^vii^—Pd5—Pd8^ix^	123.70 (7)	Pd7^viii^—Sn4—Pd5	121.64 (6)
Pd2—Pd5—Pd8^ix^	117.48 (10)	Pd1^xviii^—Sn4—Pd5	127.34 (9)
Pd4^xiv^—Pd5—Pd8^ix^	61.79 (6)	Pd5^xv^—Sn4—Pd5	96.82 (3)
Pd7^vii^—Pd5—Pd8^ix^	178.43 (9)	Pd3^xviii^—Sn4—Pd5	95.86 (4)
Sn4^xii^—Pd5—Pd8^ix^	54.16 (5)	Pd6—Sn5—Pd1^xvii^	148.90 (6)
Pd6^xii^—Pd5—Pd8^ix^	104.12 (5)	Pd6—Sn5—Pd4^viii^	67.13 (9)
Pd6—Pd5—Pd8^ix^	76.31 (9)	Pd1^xvii^—Sn5—Pd4^viii^	108.52 (4)
Sn2—Pd5—Sn4	100.70 (8)	Pd6—Sn5—Pd7	100.78 (4)
Sn5^xii^—Pd5—Sn4	79.87 (5)	Pd1^xvii^—Sn5—Pd7	66.73 (10)
Sn3^ix^—Pd5—Sn4	78.91 (8)	Pd4^viii^—Sn5—Pd7	149.46 (6)
Sn3^vii^—Pd5—Sn4	100.63 (6)	Pd6—Sn5—Pd5^xv^	64.05 (8)
Pd2—Pd5—Sn4	58.94 (5)	Pd1^xvii^—Sn5—Pd5^xv^	143.48 (12)
Pd4^xiv^—Pd5—Sn4	121.17 (9)	Pd4^viii^—Sn5—Pd5^xv^	62.38 (5)
Pd7^vii^—Pd5—Sn4	53.93 (4)	Pd7—Sn5—Pd5^xv^	139.46 (6)
Sn4^xii^—Pd5—Sn4	179.39 (14)	Pd6—Sn5—Pd8^viii^	87.99 (6)
Pd6^xii^—Pd5—Sn4	126.50 (10)	Pd1^xvii^—Sn5—Pd8^viii^	64.94 (5)
Pd6—Pd5—Sn4	53.18 (6)	Pd4^viii^—Sn5—Pd8^viii^	61.40 (4)
Pd8^ix^—Pd5—Sn4	125.24 (13)	Pd7—Sn5—Pd8^viii^	91.30 (5)
Sn4—Pd6—Sn2	110.61 (9)	Pd5^xv^—Sn5—Pd8^viii^	123.30 (7)
Sn4—Pd6—Sn5	109.00 (8)	Pd6—Sn5—Pd3^xvii^	140.17 (7)
Sn2—Pd6—Sn5	139.69 (6)	Pd1^xvii^—Sn5—Pd3^xvii^	61.14 (6)
Sn4—Pd6—Pd9	154.46 (4)	Pd4^viii^—Sn5—Pd3^xvii^	144.02 (11)
Sn2—Pd6—Pd9	73.65 (6)	Pd7—Sn5—Pd3^xvii^	62.41 (6)
Sn5—Pd6—Pd9	73.96 (6)	Pd5^xv^—Sn5—Pd3^xvii^	104.05 (4)
Sn4—Pd6—Pd4	146.07 (6)	Pd8^viii^—Sn5—Pd3^xvii^	125.70 (8)
Sn2—Pd6—Pd4	73.51 (6)	Pd6—Sn5—Pd7^ix^	80.39 (6)
Sn5—Pd6—Pd4	69.63 (6)	Pd1^xvii^—Sn5—Pd7^ix^	121.86 (8)
Pd9—Pd6—Pd4	59.38 (5)	Pd4^viii^—Sn5—Pd7^ix^	123.48 (8)
Sn4—Pd6—Sn3^viii^	88.76 (5)	Pd7—Sn5—Pd7^ix^	78.91 (5)
Sn2—Pd6—Sn3^viii^	81.94 (5)	Pd5^xv^—Sn5—Pd7^ix^	62.05 (6)
Sn5—Pd6—Sn3^viii^	91.82 (6)	Pd8^viii^—Sn5—Pd7^ix^	163.04 (5)
Pd9—Pd6—Sn3^viii^	116.71 (6)	Pd3^xvii^—Sn5—Pd7^ix^	61.63 (7)
Pd4—Pd6—Sn3^viii^	57.88 (6)	Pd6—Sn5—Pd4	56.77 (6)
Sn4—Pd6—Sn3^ix^	82.84 (4)	Pd1^xvii^—Sn5—Pd4	94.62 (5)
Sn2—Pd6—Sn3^ix^	94.90 (8)	Pd4^viii^—Sn5—Pd4	95.82 (4)
Sn5—Pd6—Sn3^ix^	97.05 (8)	Pd7—Sn5—Pd4	56.10 (7)
Pd9—Pd6—Sn3^ix^	71.64 (3)	Pd5^xv^—Sn5—Pd4	120.73 (11)
Pd4—Pd6—Sn3^ix^	131.01 (6)	Pd8^viii^—Sn5—Pd4	59.62 (4)
Sn3^viii^—Pd6—Sn3^ix^	169.38 (7)	Pd3^xvii^—Sn5—Pd4	118.48 (10)
Sn4—Pd6—Pd5^xv^	62.76 (5)	Pd7^ix^—Sn5—Pd4	103.49 (4)

Symmetry codes: (i) *x*−1, *y*−1, *z*; (ii) *x*−*y*, −*y*+1, −*z*+1/3; (iii) *x*−*y*, −*y*, −*z*+1/3; (iv) −*x*+*y*, −*x*, *z*+1/3; (v) *x*−*y*−1, −*y*, −*z*+1/3; (vi) *x*−1, *y*, *z*; (vii) *x*, *y*−1, *z*; (viii) *x*−*y*+1, −*y*+1, −*z*+1/3; (ix) *y*, *x*, −*z*; (x) *y*−1, *x*−1, −*z*; (xi) −*y*, *x*−*y*, *z*−1/3; (xii) *y*, *x*−1, −*z*; (xiii) −*x*+*y*+1, −*x*+1, *z*+1/3; (xiv) −*y*+1, *x*−*y*, *z*−1/3; (xv) *y*+1, *x*, −*z*; (xvi) *x*, *y*+1, *z*; (xvii) *x*+1, *y*+1, *z*; (xviii) *x*+1, *y*, *z*.
